# Early Behavioral Markers of Loss of Financial Capacity

**DOI:** 10.1001/jamanetworkopen.2025.15894

**Published:** 2025-06-13

**Authors:** Anna Trendl, Alexander Anwyl-Irvine, Lara Vomfell, Emma Abbey, Neil Stewart, David Atkins, David J. Llewellyn, John Gathergood, David Leake

**Affiliations:** 1 Behavioural Science, Lloyds Banking Group, London, United Kingdom; 2 Warwick Business School, University of Warwick, Coventry, United Kingdom; 3 UK AI Security Institute, London, United Kingdom; 4 Gloucestershire Health and Care NHS Foundation Trust, Gloucester, United Kingdom; 5 University of Exeter Medical School, Exeter, United Kingdom; 6 School of Economics, University of Nottingham, Nottingham, United Kingdom

## Abstract

**Importance:**

Many conditions, including neurodegenerative diseases and psychiatric disorders, can impair financial decision-making in older age. Although banking data offer rich insights, they have not yet been leveraged to understand how the loss of financial capacity is associated with financial behaviors.

**Objective:**

To describe the behavioral indicators of financial capacity loss and the association of financial capacity loss with financial vulnerability.

**Design, Setting, and Participants:**

In this case-control study, banking data recorded by a major UK bank between January 1, 2009, and April 21, 2023, were used to compare the financial outcomes of a group of 16 742 donors of power of attorney (PoA) registrations with a “loss of financial capacity” marker and a control group of 50 226 individuals with no reported financial capacity loss that matched the demographic and socioeconomic characteristics of the donor group 10 years prior to their PoA registration. Group differences in financial outcomes were examined in the 10-year period leading up to the PoA registration. Analysis took place between December 2023 and December 2024.

**Exposures:**

Bank registrations of PoAs recorded between 2019 and 2023 for which the attorney reported that the donor lost financial capacity.

**Main Outcome Measures:**

A broad range of 344 financial measures capturing day-to-day transactional activity (eg, spending on travel and hobbies) and other financial behaviors (eg, online banking logins).

**Results:**

The group of donors of PoA registrations with a “loss of financial capacity” marker comprised 16 742 individuals (mean [SD] age, 72.8 [8.5] years; 10 285 women [61.4%]), and the control group comprised 50 226 individuals (mean [SD] age, 72.7 [8.2] years; 30 657 women [61.0%]). During the 5 years prior to PoA registration, compared with the control group, donors were increasingly less likely to spend on everyday activities (clothing [difference, −9.1 percentage points (pp); 95% CI, −10.0 to −8.3 pp], travel [eg, hotels; difference, −9.6 pp; 95% CI, −10.5 to −8.8 pp], hobbies [eg, gardening; difference, −7.9 pp; 95% CI, −8.8 to −7.1 pp]) and more likely to spend on items associated with increased time at home (eg, household gas and electricity bills [difference, 5.1 pp; 95% CI, 4.6-5.7 pp]). Signs of heightened financial vulnerability in the donor group compared with the control group included an increase in the frequencies of PIN (personal identification number) reset requests (difference, 0.002 [95% CI, 0.002-0.003]), fraud cases (eg, animal charity difference, 0.0003 [95% CI, 0.0002-0.0003]), and lost or stolen credit or debit cards reported (difference, 0.005 [95% CI, 0.004-0.006]) and increased spending on charity (difference, 1.1 pp [95% CI, 0.5-1.7 pp]). In addition, the donor group exhibited reduced attention to finances compared with the control group via decreased online banking activity (difference in number of monthly online banking logins, −1.0 [95% CI, −1.1 to −0.8]).

**Conclusions and Relevance:**

This study highlighted the financial behaviors and vulnerabilities associated with declining financial capacity, such as a decrease in activity across multiple domains of daily life. These findings illustrate how banking data can reveal early behavioral signs and financial harms associated with financial capacity loss.

## Key Points

**Question:**

Can banking data reveal early symptoms of declining financial capacity and associated financial vulnerabilities?

**Findings:**

In this case-control study, the financial behaviors of 16 742 donors of power of attorney bank registrations with a “loss of financial capacity” marker were compared with a matched control group of 50 226 individuals. The analysis of 344 transactional and nontransactional financial indicators revealed that in the 10 years before power of attorney bank registration due to loss of financial capacity, the donor group reduced their everyday activities, spent more time at home, and showed signs of increased financial vulnerability compared with the control group.

**Meaning:**

This study suggests that banking data can reveal behavioral markers and financial vulnerabilities associated with the loss of financial capacity.

## Teaser

This case-control study uses banking data to describe the behavioral indicators of financial capacity loss and their association with financial vulnerability.

## Introduction

In the UK, power of attorney (PoA) is a legal mechanism by which an individual (the donor) appoints someone (an attorney) to make financial decisions on their behalf if the donor loses mental capacity.^1^ Alzheimer disease and related dementias (ADRD) is considered the single biggest cause of mental incapacity among older adults.^2^ In the UK, more than a million people aged 65 years or older have ADRD in 2025; this number is expected to exceed 1.4 million by 2040.^3^ Several currently incurable diseases fall under the umbrella term *ADRD*,^4^ all characterized by the progressive deterioration of cognitive functioning relating to memory, thinking, socializing, and the ability to perform everyday tasks.

Financial management skills are among the first instrumental activities of daily living to worsen with ADRD.^5^ Recent research has explored the association of ADRD-related cognitive decline with financial mismanagement, overconfidence, and financial exploitation.^6,7,8,9^ Studies in this area have typically used consumer credit files, survey data, and medical data to quantify the association of cognitive decline with credit scores^10,11,12^ and wealth^13,14^ that can be identified up to 6 years prior to diagnosis. Recently, checking account statements have been used to show that early memory loss is associated with increases in excess spending.^15^

Although these studies reveal important evidence about the association between cognitive decline and financial well-being, the markers of cognitive decline across a wider spectrum of financial behaviors remain unexplored. Banking data, which hold large amounts of granular information about the everyday behaviors of millions of people, have emerged as a promising, yet underused, resource for this purpose. Financial data, such as high-frequency transactions, provide unique insight into changes in instrumental activities of daily living categories such as shopping, transportation, communication, and managing finances,^16^ as well as indicators of financial vulnerability.

Here, we aim to address this gap in the literature by conducting a large-scale retrospective analysis of banking records from a major UK retail bank linked with PoA registrations due to a loss of financial capacity. By examining the temporal evolution of a comprehensive set of 344 financial behavioral indicators in the decade preceding the PoA enactment, we describe how the loss of capacity may manifest in financial data and the association of the loss of capacity with financial well-being.

## Methods

### Study Design and Setting

We conducted a case-control study using deidentified banking records including PoA registrations recorded between January 1, 2009, and April 21, 2023, by one of the UK’s largest retail banks.^17^ Setting up a PoA aims to ensure that, should the donor lose financial capacity in the future, the attorney can gain authority over the donor’s finances by registering the PoA with the donor’s bank. The PoA can be set up only when the donor still has capacity. When the PoA is registered with the bank by the attorney, they are asked to indicate the donor’s degree of financial capacity loss: no capacity loss, losing capacity, or loss of capacity. Our analysis of deidentified banking data formed part of Lloyds Banking Group’s customer vulnerability and support strategy and was approved by Lloyds’ Banking Group’s privacy risk and impact assessment committee. On opening an account, customers consented to the use of their anonymized data for research purposes as stated in the bank’s data privacy notice. As the study uses only existing anonymized records, it was exempted from further review under the University of Nottingham’s Research Ethics protocols. This study followed the Strengthening the Reporting of Observational Studies in Epidemiology (STROBE) reporting guideline for case-control research.

Using this indicator, our objective was to identify financial markers associated with financial capacity loss in the decade preceding PoA registration by comparing 2 groups: donors of registered PoAs for which the attorney told the bank that the donor had lost financial capacity by the time of registration (loss of financial capacity [LFC] group) and a matched control group without known LFC.

### Participants and Sample Matching

For both groups, we defined the following sample inclusion criteria: over the individual-specific 10-year analysis period, the individual must (1) have held a current checking account; (2) had a mean of at least 6 monthly transactions within each year across their current checking and credit card accounts; (3) have resided in England; (4) have been between 40 and 90 years of age at the start of the analysis period; and (5) have not held a joint current checking account with another individual in the sample. The analysis period for the LFC sample is defined as the 10-year period preceding the month of their PoA registration date (n = 16 742). The PoA registration dates range from January 2019 to April 2023.

We constructed a control group to match key demographic characteristics of the LFC group at the beginning of the 10-year period. First, starting with an initial pool of 15 720 164 control candidates, we iteratively applied requirements derived from the inclusion criteria, reducing the control candidate sample to 637 964. We then randomly assigned a PoA registration date to all control candidates, sampled from the distribution of LFC registration dates. With the exact analysis period now defined for all control candidates, we then restricted this control sample to individuals who met our specified full inclusion criteria (n = 158 550). The sample selection process for the LFC and control samples are shown in eFigures 1 and 2 in Supplement 1, respectively.

Finally, we then performed propensity score matching^18,19,20,21^ with the following matching variables: PoA registration month, gender, age, median monthly credit turnover (sum of all credit to the individual’s account, excluding interaccount transfers and refunds; proxy for income), median monthly number of transactions, and deprivation rank^22^ of the geographic area where the individual lived. These variables were calculated during the month when our analysis period started (10 years before PoA registration month), with the credit turnover and monthly number of transactions calculated using data from the first year of the analysis period. Each LFC individual was matched to 3 control members with the closest propensity score using the MatchIt package, version 4.5.5 in R, version 4.3.0 (R Project for Statistical Computing).^23^

This process yielded a final control sample of 50 226 individuals and a combined sample of 66 968 individuals with identical matching variable distributions (eFigures 3 and 4 in Supplement 1). Our sample selection process ensured complete and continuous data across all matching variables during the entire analysis period.

The propensity score matching results are shown in eTable 1 in Supplement 2. The Table shows the descriptive statistics for these matching variables across the samples,^24^ while eTable 2 in Supplement 2 shows these statistics split by gender.

**Table.** Demographic Characteristics of the LFC and Control Groups

Characteristic^a^	LFC group (n = 16 742)	Control group (n = 50 226)
Period start date, mean	May 1, 2011	April 28, 2011
Age, mean (SD), y^b^	72.8 (8.5)	72.7 (8.2)
Gender, No. (%)^b^		
Male	6457 (38.6)	19 569 (39.0)
Female	10 285 (61.4)	30 657 (61.0)
Joint account, No. (%)	10 305 (61.6)	31 856 (63.4)
Deprivation rank, mean (SD)^b^^,^^c^	18 961.3 (9025.7)	18 945.1 (9011.7)
Credit turnover, mean (SD), £^d^^,^^e^	1852.1 (2615.1)	1842.1 (2079.8)
Transactional activity, mean (SD), No./y^d^	23.2 (14.4)	23.3 (14.5)

Abbreviation: LFC, loss of financial capacity.

^a^ With the exception of joint account holding, these variables were used in the propensity score matching.

^b^ Mean values were calculated from individual-level values in the first month of the analysis period. The analysis period is defined as the 10-year period before the actual (LFC group) or randomly assigned (control group) power of attorney registration date.

^c^ Deprivation rank: 1 represents the most deprived area and 32 844 represents the least deprived area (the number of Lower Super Output Areas in England) in 2019.^24^

^d^ Mean values were derived from individual-level median values calculated from the first year of the analysis period.

^e^ Credit turnover indicates the monthly sum of payments to the accounts in British pounds, excluding interaccount transfers and refunds. There is no theoretical maximum to credit turnover; the minimum is 0. Here we report the mean of the individual-level median of credit turnover in the first year of the analysis. In our sample, the maximum of this median was £220 728 (conversion rate, £1 = US $1.34).

### Data Sources and Measures

The financial data of these 66 968 individuals covering the individual-specific 10-year period were retrieved, encompassing transaction data and other nontransactional measures (eg, online banking activity). Records ranged between January 2009 and April 2023.

A transaction was defined as any credit or debit that occurred on a personal current checking or credit card account, including electronic transfers, online transactions, and cash (ATM [automated teller machine]) withdrawals. Of the 411 million debit and credit card transactions retrieved, most (98%) were classified by the banks’ internal transaction classification system into 464 transaction categories. We kept only transaction categories in which at least 1% of the overall sample had a transaction during our analysis period, resulting in 303 transaction categories. For our subsequent analyses involving transaction data, we converted monthly transaction count data into a binary format to capture the presence or absence of a transaction in a specific month.

Additional nontransactional data features (n = 41), a mix of binary and frequency indicators, were also extracted, including product holdings, overdraft use, interactions with the bank, fraud, and lost or stolen cards. The full list of 344 financial variables constructed can be found in eTable 3 in Supplement 2.

### Statistical Analysis

#### Group Differences

Analysis took place between December 2023 and December 2024. To formally test the difference in activity between the LFC and control groups over a certain period across 344 financial measures, χ^2^ tests were used to test the equality of group proportions with at least 1 transaction in each transaction category, and *t* tests were used to test the equality of group mean values within nontransactional categories. To account for multiple comparisons, we used a permutation approach with 1000 iterations.^25^

#### Temporal Evolution of Group Differences

A linear regression model with an ordinary least-squares estimation was used to compare financial behaviors across groups and time. Due to computational limitations, we included only transactional categories in this analysis, in which at least 15% of the overall sample had at least 1 transaction (n = 148) during the analysis period; therefore, the overall number of measures (transactional and nontransactional) included in this analysis was 189 (for a full list, see eTable 3 in Supplement 2). The analysis generated a 2-dimensional matrix of *t* statistics, representing the temporal evolution of group differences relative to the PoA registration date for each measure.

Our aim was to identify persistent temporal clusters of group differences in our set of measures, while controlling for multiple comparisons. We first selected temporal *t* statistics cluster candidates that exceeded 2.94 for transactional categories and 1.96 for nontransactional categories. These thresholds were selected to provide us with a reasonable number of cluster candidates for the 2 types of measures; they are not our chosen α level, nor do they affect the validity of the statistical test.^26,27^

Next, to effectively control for multiple comparisons through the familywise error rate, we used a Monte Carlo simulation method by generating joint null distributions of the largest absolute cluster sums through 1000 permutation iterations^27,28,29,30,31^ and retained only candidate clusters significant at *P* < .001. All *P* values were from 2-sided tests; analyses were conducted in Python, version 3.12.2 (Python Software Foundation) using the MNE package.^32^

## Results

Overall, 16 742 LFC individuals (6457 men [38.6%] and 10 285 women [61.4%]; mean [SD] age, 72.8 [8.5] years) and 50 226 control individuals (19 569 men [39.0%] and 30 657 women [61.0%]; mean [SD] age, 72.7 [8.2] years) were included in the matched sample (Table).^24^

### Characterizing Cognitive Decline Using Banking Data

**Figure 1.**
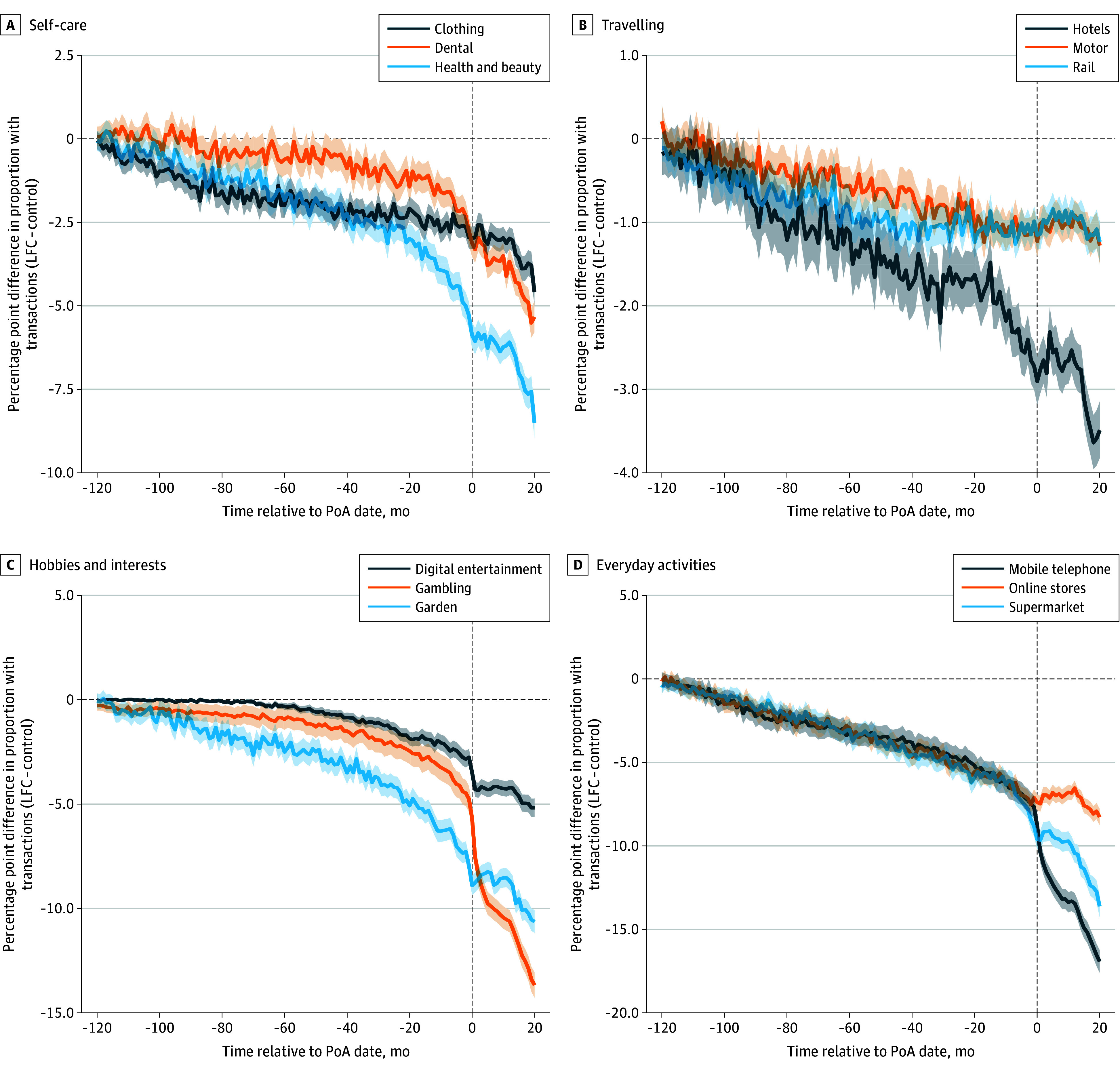
Monthly Differences in Transactional Activity Between the Loss of Financial Capacity (LFC) Group and the Control Group (Self-Care, Travelling, Hobbies and Interests, and Everyday Activities) The exact transactional categories are as follows. Self-care: Retail/Clothing/Family; Services/Health/Dental; Retail/Health/Health & Beauty. Travelling: Travel/Hotels/Other; Motor/Purchase, Maintenance and Repair/Parts; Travel/Rail/Other. Hobbies and interests: Retail/Digital Goods/Audiovisual including Books Movies Music; Hobbies & Interests/Gambling/Other; Retail/Garden/Other. Everyday activities: Communication/Mobile Phone/Other; Retail/Department and Online stores/Other; Retail/Supermarket/Discount. Shaded areas indicate 95% CIs. PoA indicates power of attorney.

**Figure 2.**
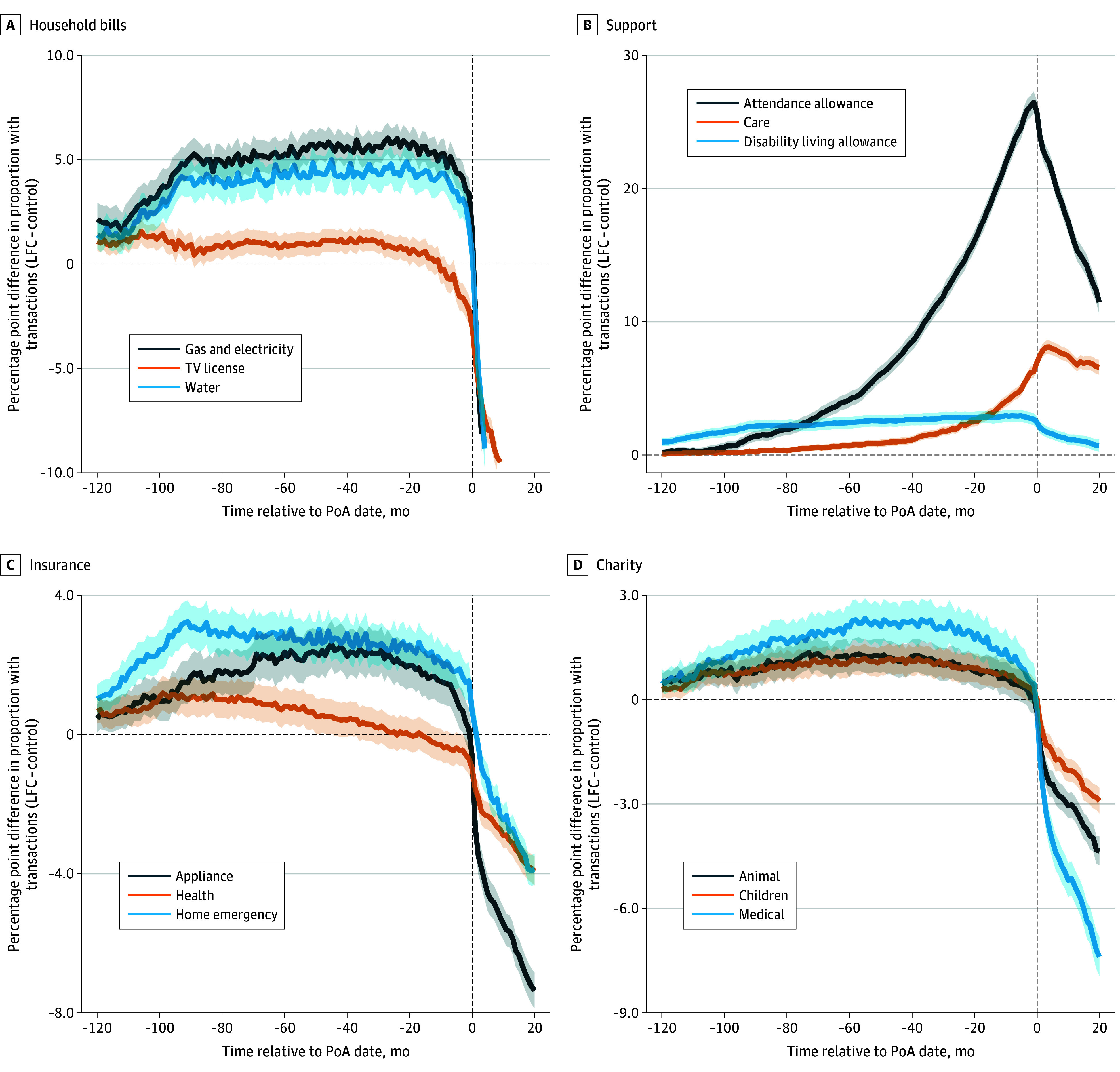
Monthly Differences in Transactional Activity Between the Loss of Financial Capacity (LFC) Group and the Control Group (Household Bills, Support, Insurance, and Charity) The exact transactional categories are as follows. House bills: Household Bills/Energy/Gas & Electricity; Household Bills/Water/Other; Household Bills/TV Licence/Other. Support: Benefits/Disability Living Allowance/Other; Benefits/Attendance Allowance/Other; Services/Health/Care. Insurance: Financial Services/Insurance/Appliance; Financial Services/Insurance/Health; Financial Services/Insurance/Home emergency. Charity: Miscellaneous/Charity/Animal; Miscellaneous/Charity/Children; Miscellaneous/Charity/Medical. Shaded areas indicate 95% CIs. PoA indicates power of attorney.

To illustrate the financial markers of potential cognitive decline, we focused on a subset of the 344 financial measures. All variables and the associated statistical tests for the periods 10 to 5 years prior to the PoA registration date and only 5 years prior to the PoA registration date can be found in eTable 3 in Supplement 2. Figure 1 and Figure 2 show the mean monthly activity difference between the LFC and control groups for selected transaction categories in the 120 months (10 years) before and 20 months after the PoA date.

We found that, compared with the control group, the LFC group had significantly lower activity in the 5-year period before the PoA date in transactional categories relating to self-care (eg, clothing; difference, −9.1 percentage points [pp] [95% CI, −10.0 to −8.3 pp]; *P* < .001), traveling (eg, hotels; difference, −9.6 pp [95% CI, −10.5 to −8.8 pp]; *P* < .001), hobbies and interests (eg, gardening; difference, −7.9 pp [95% CI, −8.8 to −7.1 pp]; *P* < .001), and everyday activities (eg, mobile phone; difference, −7.6 pp [95% CI, −8.5 to −6.7 pp]; *P* < .001) and significantly higher activity in categories relating to household bills (eg, gas and electricity; difference, 5.1 pp [95% CI, 4.6-5.7 pp]; *P* < .001), support (eg, attendance allowance [a type of benefit in the UK available to those who need help with personal care^33^]; difference, 29.8 pp [95% CI, 28.9-30.6 pp]; *P* < .001), insurance (eg, appliance; difference, 2.9 pp [95% CI, 2.1-3.7 pp]; *P* < .001), and charity (eg, animal; difference, 1.1 pp [95% CI, 0.5-1.7 pp]; *P* < .001). In all cases, these differences gradually increased in absolute terms as we moved toward the PoA registration date. These patterns were robust to the following changes in our sample construction, including joint account holding as a matching variable, changing the transactional activity threshold to 2 and 8 (instead of 6), and restricting the lower end of age range (60 years instead of 40 years), as shown in eFigures 5 to 16 in Supplement 1.

**Figure 3.**
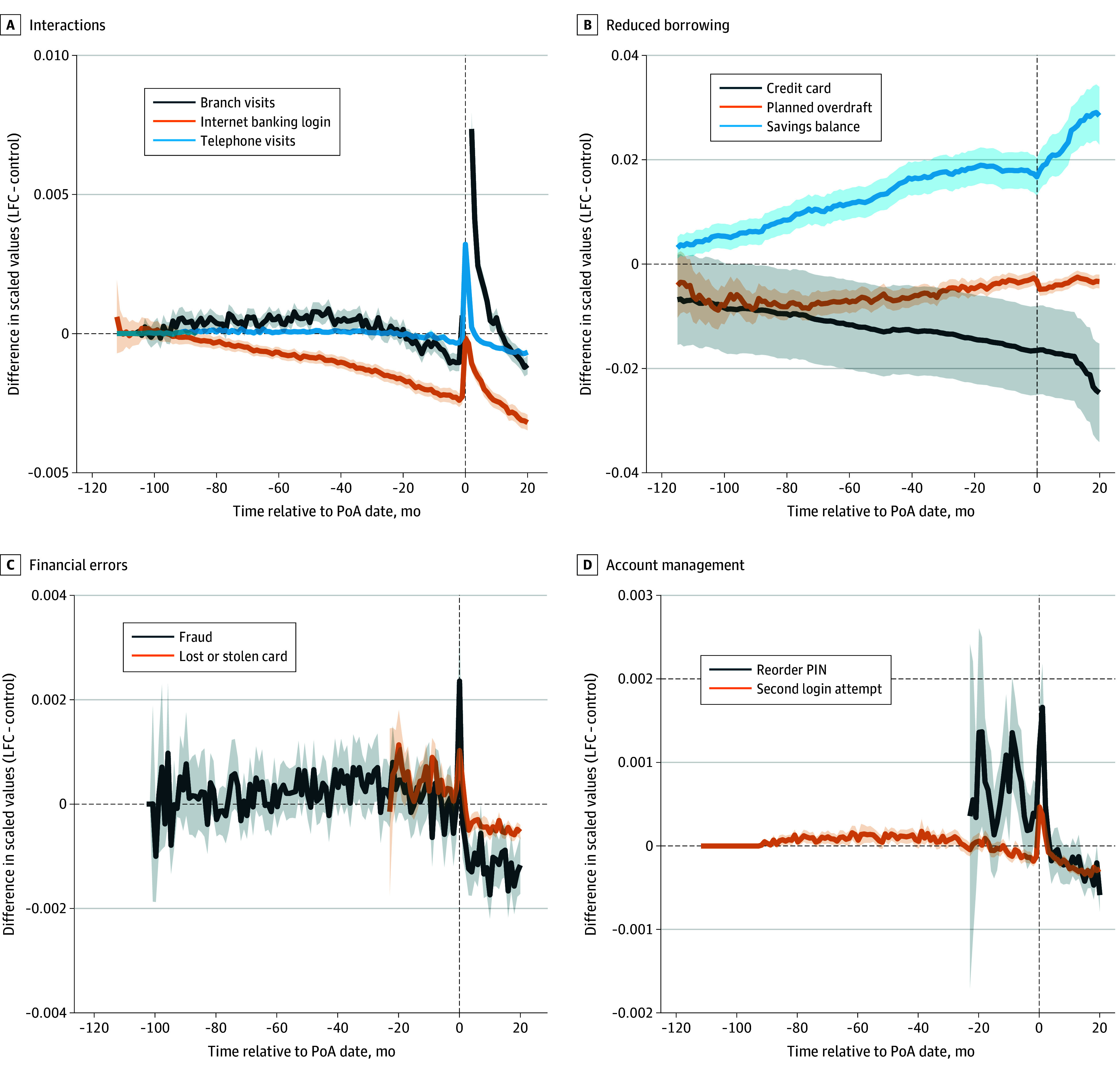
Monthly Mean of Differences Between the Loss of Financial Capacity (LFC) Group and the Control Group Calculated From Normalized Variables Planned overdraft and savings balance measures were winsorized at the 99th percentile prior to normalization. Data on lost or stolen cards, personal identification number (PIN) reorders, and reported cases of fraud are not available for the entire 10-year period. The difference in number of monthly branch visits peaked at 0.086 in the month of the power of attorney (PoA) (month 0). Shaded areas indicate 95% CIs.

Figure 3 demonstrates group differences in individual-level mean monthly counts of nontransactional activity features across 4 areas: interactions with the bank, reduced borrowing, financial errors, and account management. Compared with the control group, in the 5-year period preceding the PoA registration date, individuals in the LFC group had a lower mean number of monthly internet banking logins (difference, −1.0 [95% CI, −1.1 to −0.8]; *P* < .001) and reported higher frequencies of fraud incidents (difference, 0.0003 [95% CI, 0.0002-0.0003]; *P* < .001), lost or stolen cards (difference, 0.005 [95% CI, 0.004-0.006]; *P* < .001), and PIN (personal identification number) reset requests (difference, 0.002 [95% CI, 0.002-0.003]; *P* < .001). The mean (SD) material loss to fraud in our sample was £505 (£3352) (conversion rate, £1 = US $1.34).

### Temporal Pattern of Group Differences

**Figure 4.**
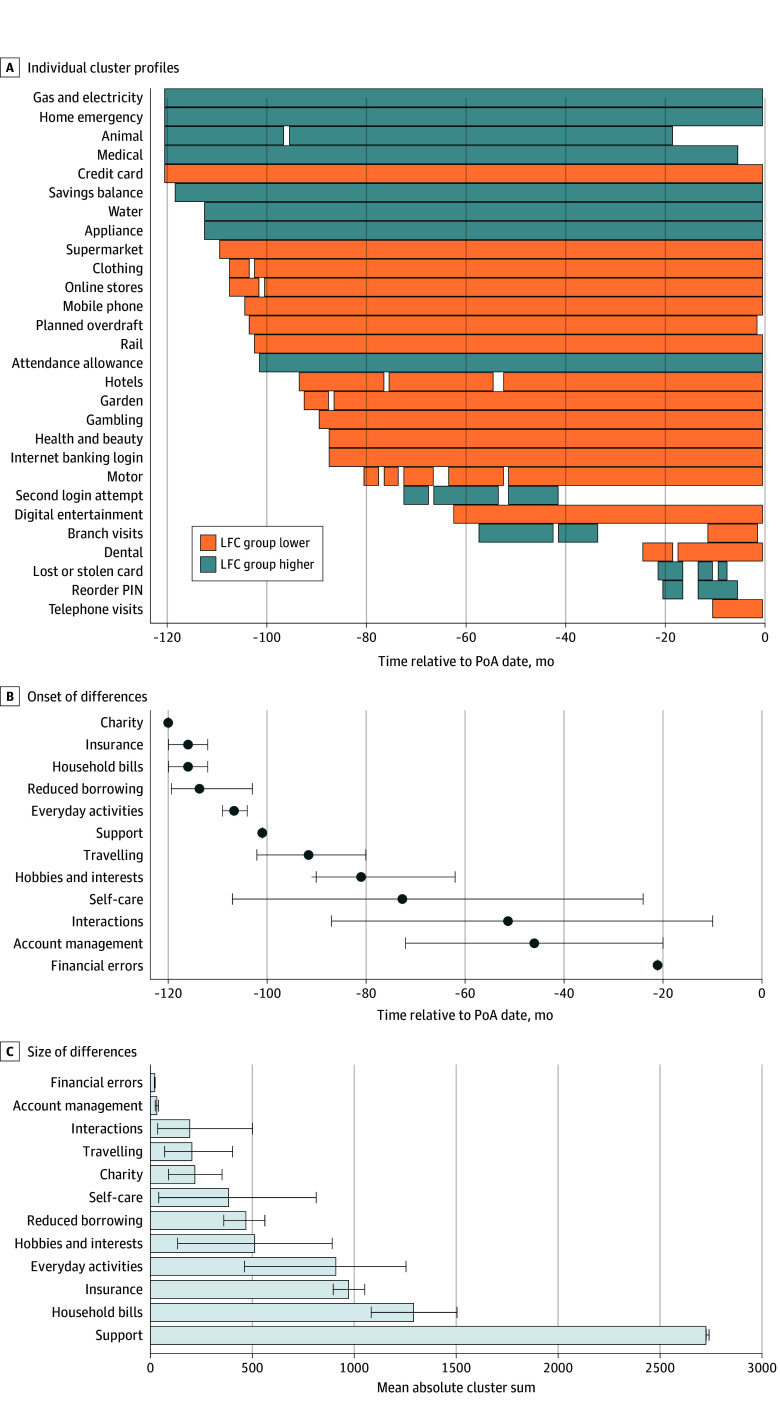
Temporal Pattern and Size of Differences Between the Loss of Financial Capacity (LFC) Group and the Control Group Based on Temporal Clustering Analysis A, Significant clusters over time by the direction of difference. B and C, The mean earliest starting point and the mean absolute sum of *t* values from significant clusters, respectively. Error bars indicate bootstrapped 95% CIs. Only transactional categories were included in which at least 15% of the overall sample had at least 1 transaction in our analysis period. PIN indicates personal identification number; and PoA, power of attorney.

Figure 4 shows that, on average, differences in financial indicators appeared in the following order: increase in charity, insurance, household bills spend 10 years before registration; reduction in borrowing activity and spend on everyday activities 9 years before registration; increased support and reduced traveling 8 years before registration; reduced spend on hobbies and interests 7 years before registration; reduced spend on self-care 6 years before registration; and reduced interactions with the bank and account management activity 4 years before registration and increased financial errors 2 years before registration.

## Discussion

By comparing the financial activity of individuals who have lost their financial capacity with those of a matched control group that did not report financial capacity loss, we found important behavioral differences between the 2 groups in the period preceding the PoA registration. In the LFC group, differences indicate lower physical and mental activity and increased financial vulnerability and time spent at home. More frequent household bills may result from forgetting to turn off appliances or needing to use the washing machine more often due to continence issues.^34^

Previous research has documented the association between loss of capacity and increased risk of financial exploitation.^9,35,36^ We identified several findings that support this association. First, our results indicate that the LFC group was slightly more likely to report fraud in the analysis period, and this difference significantly increased in the month of the PoA, suggesting that in some proportion of cases, fraud was either the trigger for the PoA registration or past fraud was discovered once the attorney gained access to the donor’s account. Given that currently only 15% of the UK adult population have a PoA in place,^37^ this finding raises a significant concern about the scale of undetected fraud harming those with declining financial capacity and no PoA protection. Although the baseline-reported fraud frequency was relatively small, the material loss can be significant in each of these cases (the mean [SD] loss in our sample was £505 [£3352]). In addition, our frequency estimate is most likely an underestimation because individuals with declining financial capacity are more vulnerable to fraud^36^ while at the same time they are less likely to be able to recognize that they have been a target of fraud,^35^ resulting in disproportionately lower reporting rates.

Second, we found that the LFC group exhibited consistently higher spending activity toward different types of charities compared with the control group during our analysis period. Although this finding is in line with previous reports of an association between early Alzheimer disease symptoms and increased financial altruism,^8^ it may also be a sign of increased financial vulnerability. Due to the increased time spent at home, those with declining capacity are more likely to be exposed to cold-calling by telephone or in person,^38^ and they might forget existing direct debits and end up duplicating payments. These potentially unwanted payments may add up to a significant financial burden for the donor.

Last, we observed a significant decrease in the online banking activity of the LFC group and an increase in repeated login attempts, lost or stolen cards, and PIN reorders, suggesting that the LFC group was gradually losing the ability to monitor their finances, which in turn increased the likelihood of unwanted transactions going unnoticed. In addition to highlighting the financial vulnerability associated with losing financial capacity, this study illustrates how granular financial data can help us understand how declining financial capacity manifests in everyday behaviors. In contrast to biomarkers, cognitive and functional measures, such behavioral financial data, are already stored in large volumes by financial institutions, which have a direct interest in protecting vulnerable customers. If data privacy and individual consent considerations allow, combining these data sources could provide valuable insights into how behavioral markers vary across specific conditions (eg, Alzheimer disease vs frontotemporal dementia). Such insights may inform the development of screening strategies.

Other promising areas for future research include assessing whether capacity loss–related financial harm varies across sociodemographic groups and evaluating the effectiveness of PoA and similar safeguards in protecting individuals. These insights could play a role in shaping the development of PoA policy. Although the number of PoA registrations is steadily increasing in the UK,^37^ public awareness remains relatively low,^39^ and there are several issues identified with the current PoA system, including a costly application process,^40^ long delays,^37^ and cases of PoA abuse.^41^ Given the projected increase in the number of adults living with declining financial capacity in the UK and the association between loss of capacity and financial vulnerability, efforts to improve the current PoA system could yield significant societal benefits.

### Limitations

Our study has a number of limitations. With no access to medical diagnoses, we must rely on proxy information given about the donor’s financial capacity by the attorney. Although attorneys have no incentive to misreport the donor’s financial capacity at the point of registration, such self-reported data are inherently subject to some degree of inaccuracy.

Because most UK adults do not have a PoA, some individuals in the control group may have cognitive decline. Individuals with joint accounts likely receive support from the coholder, reducing their need for a PoA. In addition, due to cost, PoAs may be more common among those with higher socioeconomic status. Therefore, our results may not be generalizeable to individuals with a lower socioeconomic status or those with existing support. However, because individuals with a lower socioeconomic status are more vulnerable to ADRD-associated financial harm,^10^ we are potentially underestimating the degree of cognitive decline–associated financial harm.

Due to data availability, we had to restrict our analysis period to 10 years, apply sample criteria, and exclude individuals from the sample who ceased using the bank’s services but would otherwise have been included in the LFC or control groups. The differences between the LFC and control groups found in some of the financial indicators are apparent at the beginning of the analysis period, suggesting that a longer analysis period might be required.

Finally, we cannot track cash spending. Although the popularity of cash in the UK has been decreasing steadily, older age groups are more likely to prefer this method of payment,^42^ potentially limiting the insights offered by banking data.

## Conclusions

In this case-control study, donors of registered PoAs with financial capacity loss showed a marked decrease in activity across multiple domains of daily life and heightened financial vulnerability in the 10 years before PoA registration compared with a matched control group. These findings illustrate how banking data can reveal early behavioral signs and financial harms associated with financial capacity loss.

## Notes

Supplement 1. **eFigure 1.** Loss of Financial Capacity Sample Selection Diagram **eFigure 2.** Control Sample Selection Diagram **eFigure 3.** LFC-Control Sample Matching Variable Distributions I **eFigure 4.** LFC-Control Sample Matching Variable Distributions II **eFigure 5.** Sensitivity Results for Self-Care Transactional Activity **eFigure 6.** Sensitivity Results for Travelling Transactional Activity **eFigure 7.** Sensitivity Results for Hobbies and Interests Transactional Activity **eFigure 8.** Sensitivity Results for Everyday Activities Transactional Activity **eFigure 9.** Sensitivity Results for Household Bills Transactional Activity **eFigure 10.** Sensitivity Results for Support Transactional Activity **eFigure 11.** Sensitivity Results for Insurance Transactional Activity **eFigure 12.** Sensitivity Results for Charity Transactional Activity **eFigure 13.** Sensitivity Results for Interactions Activity **eFigure 14.** Sensitivity Results for Borrowing Activity **eFigure 15.** Sensitivity Results for Financial Errors **eFigure 16.** Sensitivity Results for Account Management

Supplement 2. **eTable 1.** Propensity Score Matching Results **eTable 2.** Table 1 Sample Characteristics Split by Gender **eTable 3.** Statistical Tests for all Financial Indicators 10-5 and 5-1 Years before the PoA Registration Date

Supplement 3.
Data Sharing Statement
