# Supplementary material for: Early Behavioral Markers of Loss of Financial Capacity
Source: JAMA Netw Open. 2025 Jun 13;8(6):e2515894. doi: 10.1001/jamanetworkopen.2025.15894 (PMC12166485; doi:10.1001/jamanetworkopen.2025.15894)
Supplement: Supplement 1. — eFigure 1. Loss of Financial Capacity Sample Selection Diagram eFigure 2. Control Sample Selection Diagram eFigure 3. LFC-Control Sample Matching Variable Distributions I eFigure 4. LFC-Control Sample Matching Variable Distributions II eFigure 5. Sensitivity Results for Self-Care Transactional Activity eFigure 6. Sensitivity Results for Travelling Transactional Activity eFigure 7. Sensitivity Results for Hobbies and Interests Transactional Activity eFigure 8. Sensitivity Results for Everyday Activities Transactional Activity eFigure 9. Sensitivity Results for Household Bills Transactional Activity eFigure 10. Sensitivity Results for Support Transactional Activity eFigure 11. Sensitivity Results for Insurance Transactional Activity eFigure 12. Sensitivity Results for Charity Transactional Activity eFigure 13. Sensitivity Results for Interactions Activity eFigure 14. Sensitivity Results for Borrowing Activity eFigure 15. Sensitivity Results for Financial Errors eFigure 16. Sensitivity Results for Account Management [file jamanetwopen-e2515894-s001.pdf]

Supplementary Online Content

Trendl A, Anwyl-Irvine A, Vomfell L, et al. Early behavioral markers of loss of financial capacity. *JAMA Netw Open.* 2025;8(6):e2515894. doi:10.1001/jamanetworkopen.2025.15894

eFigure 1. Loss of Financial Capacity Sample Selection Diagram

eFigure 2. Control Sample Selection Diagram

eFigure 3. LFC-Control Sample Matching Variable Distributions I

eFigure 4. LFC-Control Sample Matching Variable Distributions II

eFigure 5. Sensitivity Results for Self-Care Transactional Activity

eFigure 6. Sensitivity Results for Travelling Transactional Activity

eFigure 7. Sensitivity Results for Hobbies and Interests Transactional Activity

eFigure 8. Sensitivity Results for Everyday Activities Transactional Activity

eFigure 9. Sensitivity Results for Household Bills Transactional Activity

eFigure 10. Sensitivity Results for Support Transactional Activity

eFigure 11. Sensitivity Results for Insurance Transactional Activity

eFigure 12. Sensitivity Results for Charity Transactional Activity

eFigure 13. Sensitivity Results for Interactions Activity

eFigure 14. Sensitivity Results for Borrowing Activity

eFigure 15. Sensitivity Results for Financial Errors

eFigure 16. Sensitivity Results for Account Management

This supplementary material has been provided by the authors to give readers additional information about their work.

eFigure 1. Loss of Financial Capacity Sample Selection Diagram

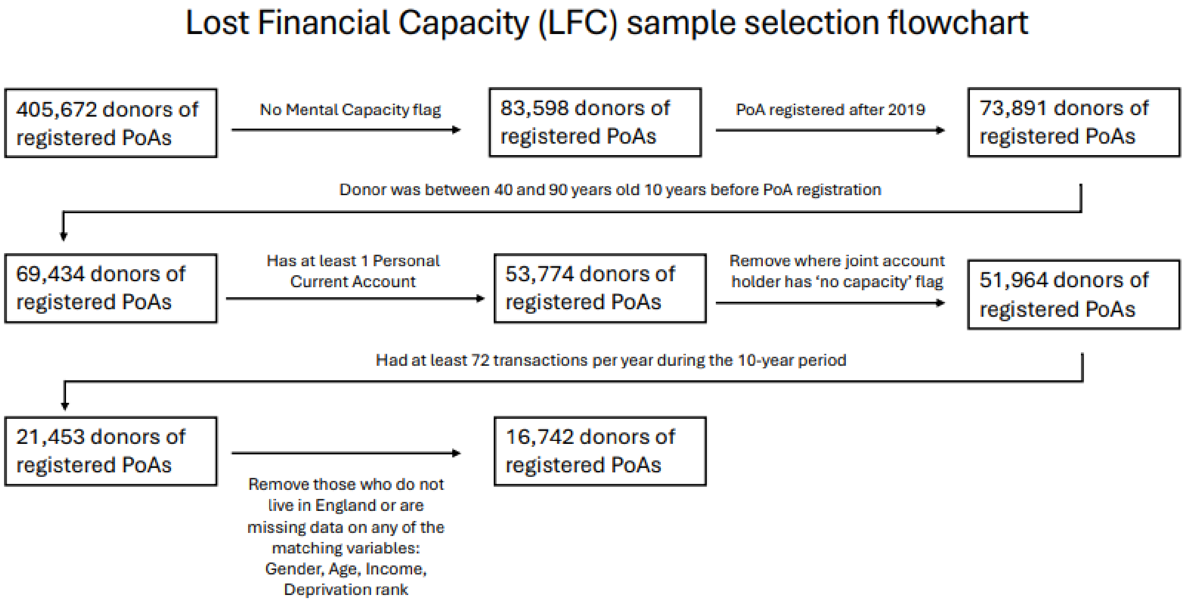

© 2025 Trendl A et al. *JAMA Network Open.*

eFigure 2. Control Sample Selection Diagram

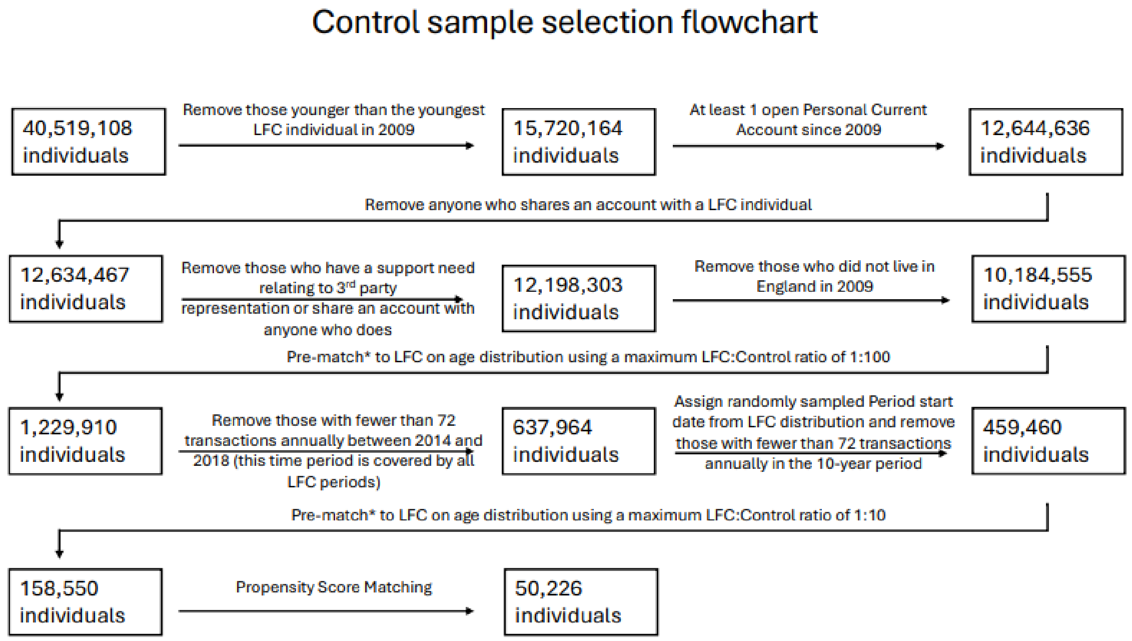

* Entails randomly sampling x individuals from the Control sample for each LFC age, to obtain an identical age distribution in the LFC and the resulting Control sample

© 2025 Trendl A et al. *JAMA Network Open.*

eFigure 3. LFC-Control Sample Matching Variable Distributions I

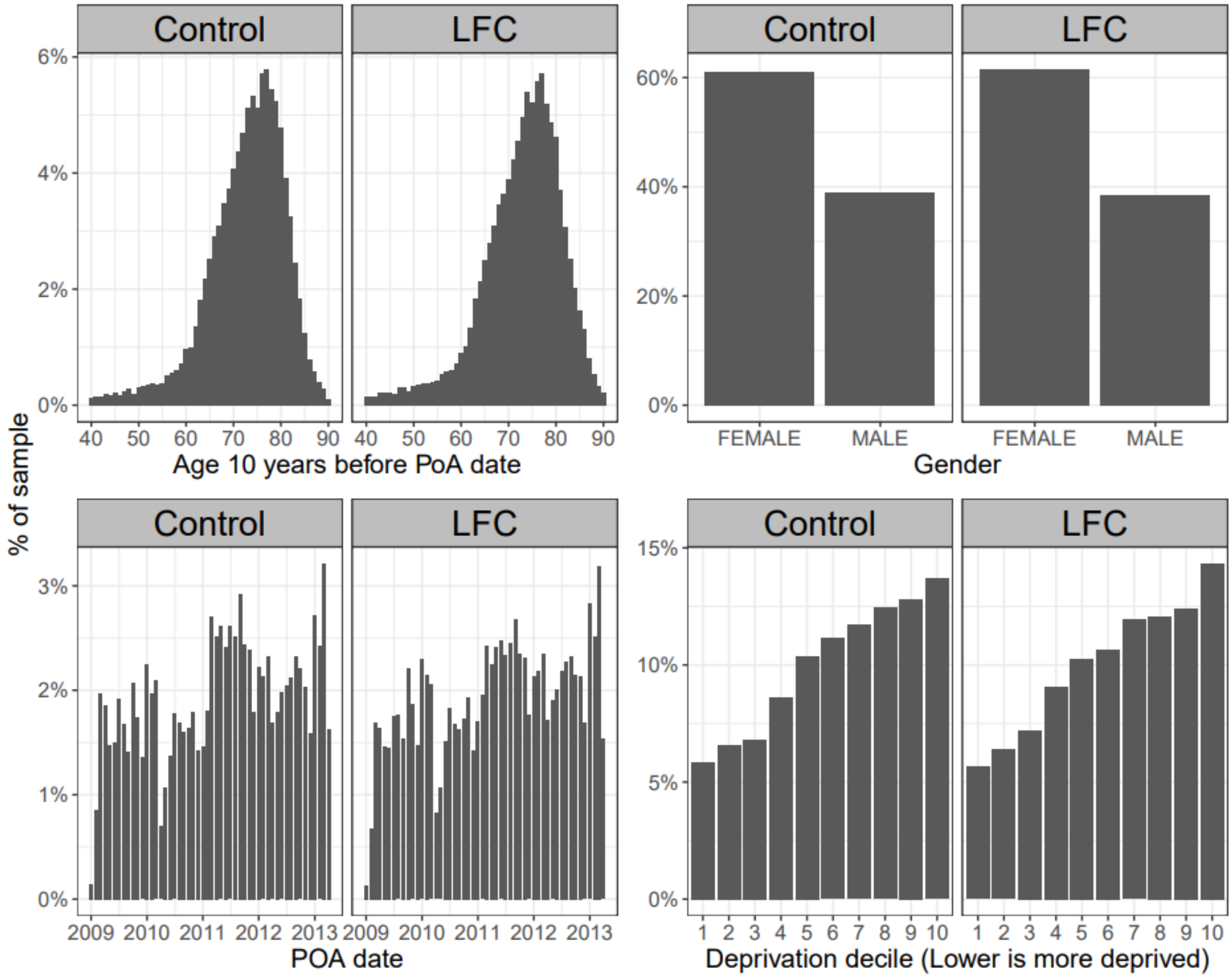

© 2025 Trendl A et al. *JAMA Network Open.*

eFigure 4. LFC-Control Sample Matching Variable Distributions II

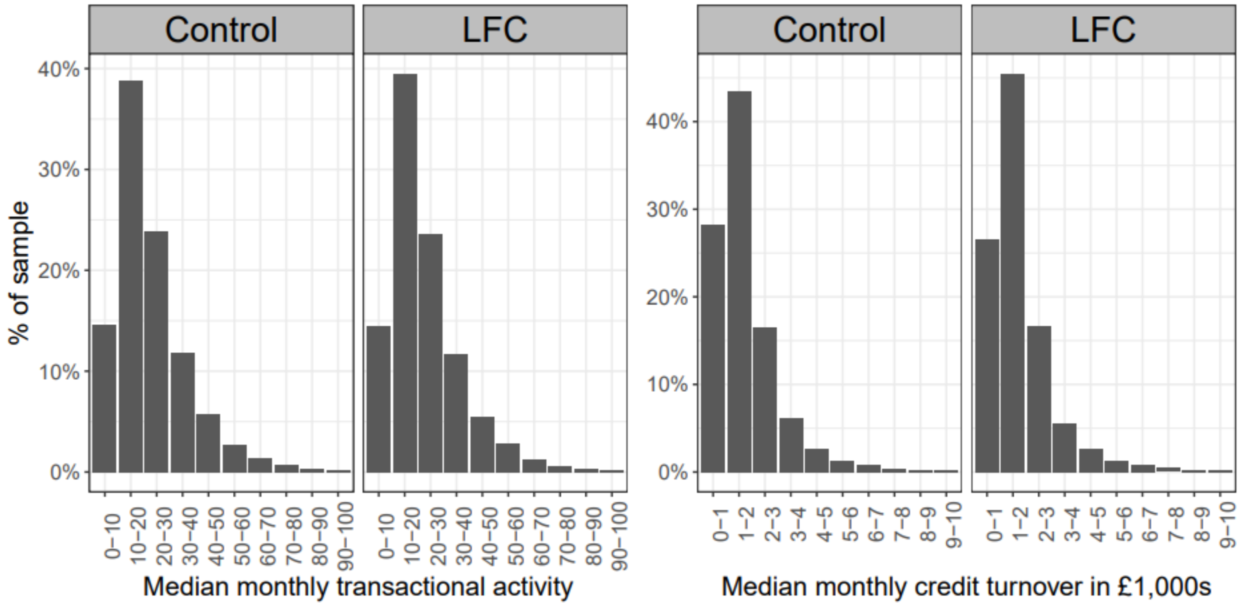

© 2025 Trendl A et al. *JAMA Network Open.*

eFigure 5. Sensitivity Results for Self-Care Transactional Activity

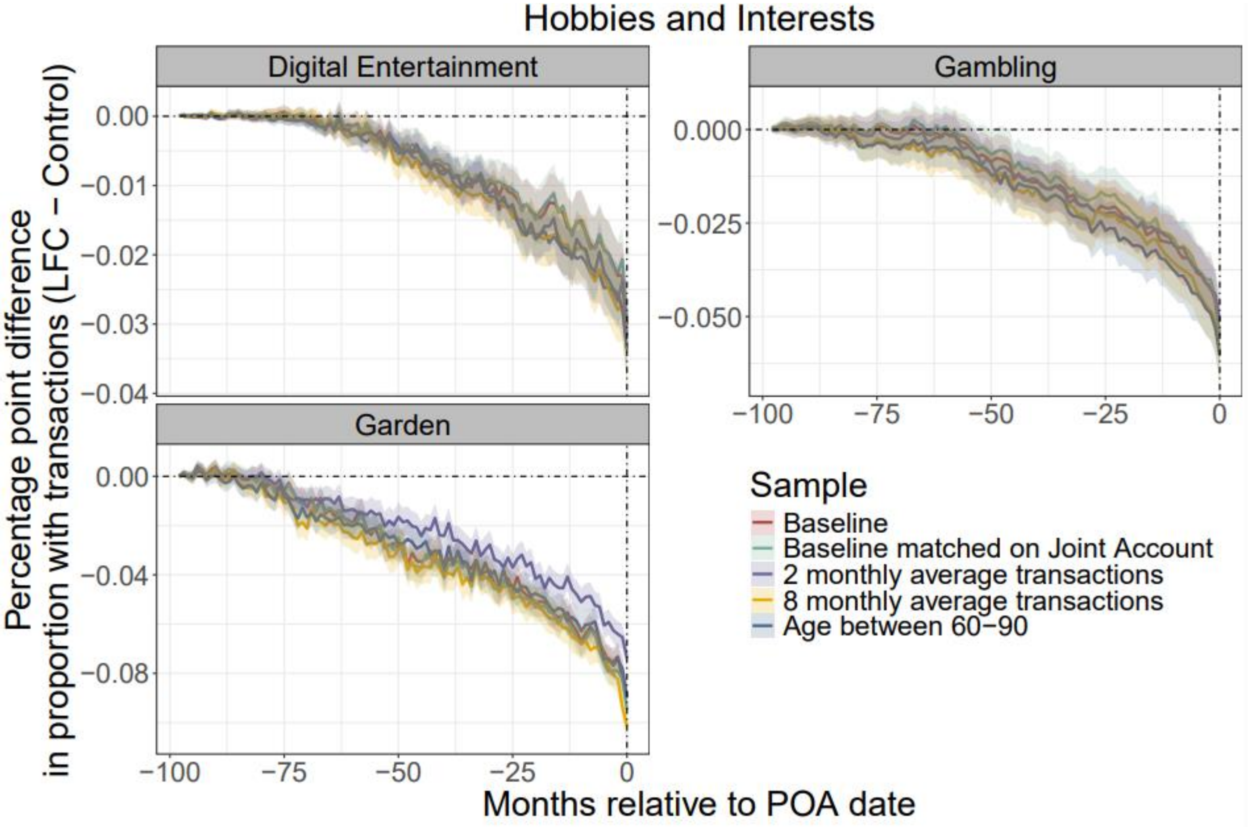

© 2025 Trendl A et al. *JAMA Network Open.*

eFigure 6. Sensitivity Results for Travelling Transactional Activity

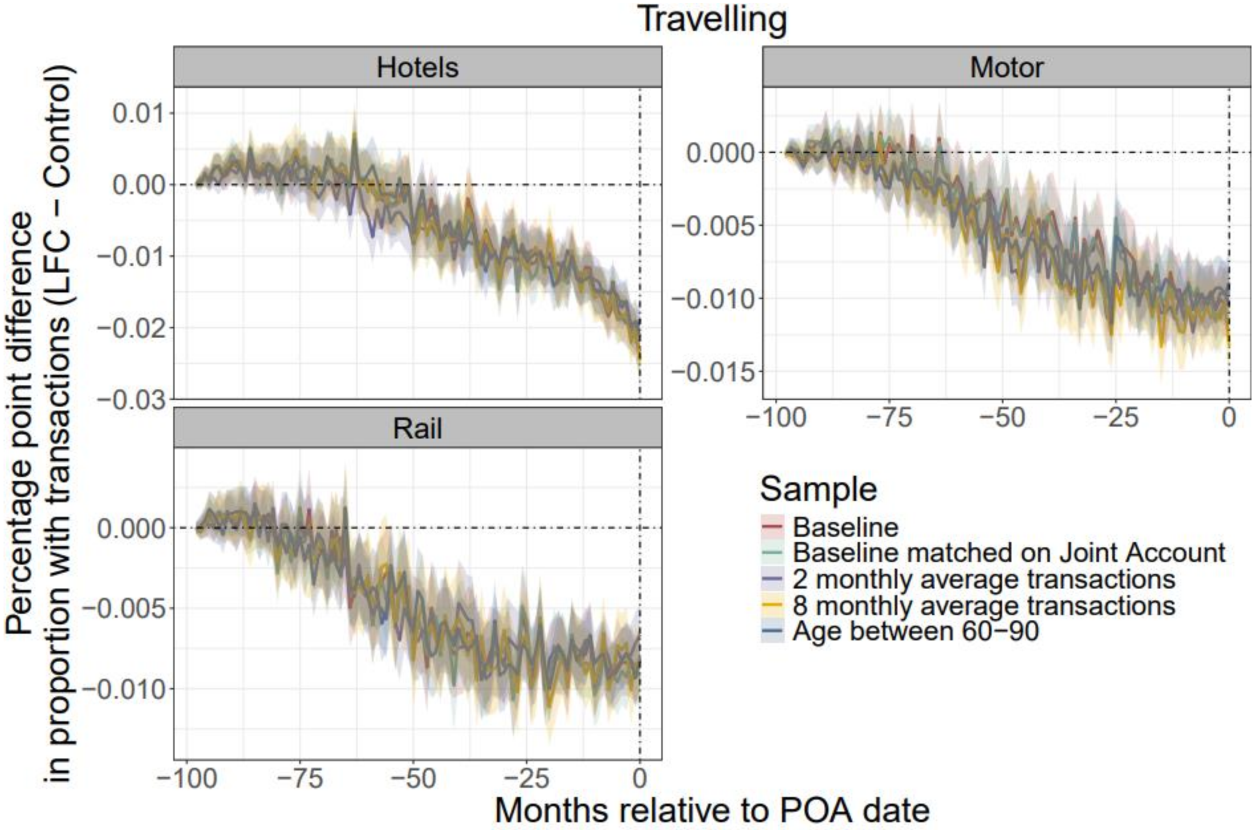

© 2025 Trendl A et al. *JAMA Network Open.*

eFigure 7. Sensitivity Results for Hobbies and Interests Transactional Activity

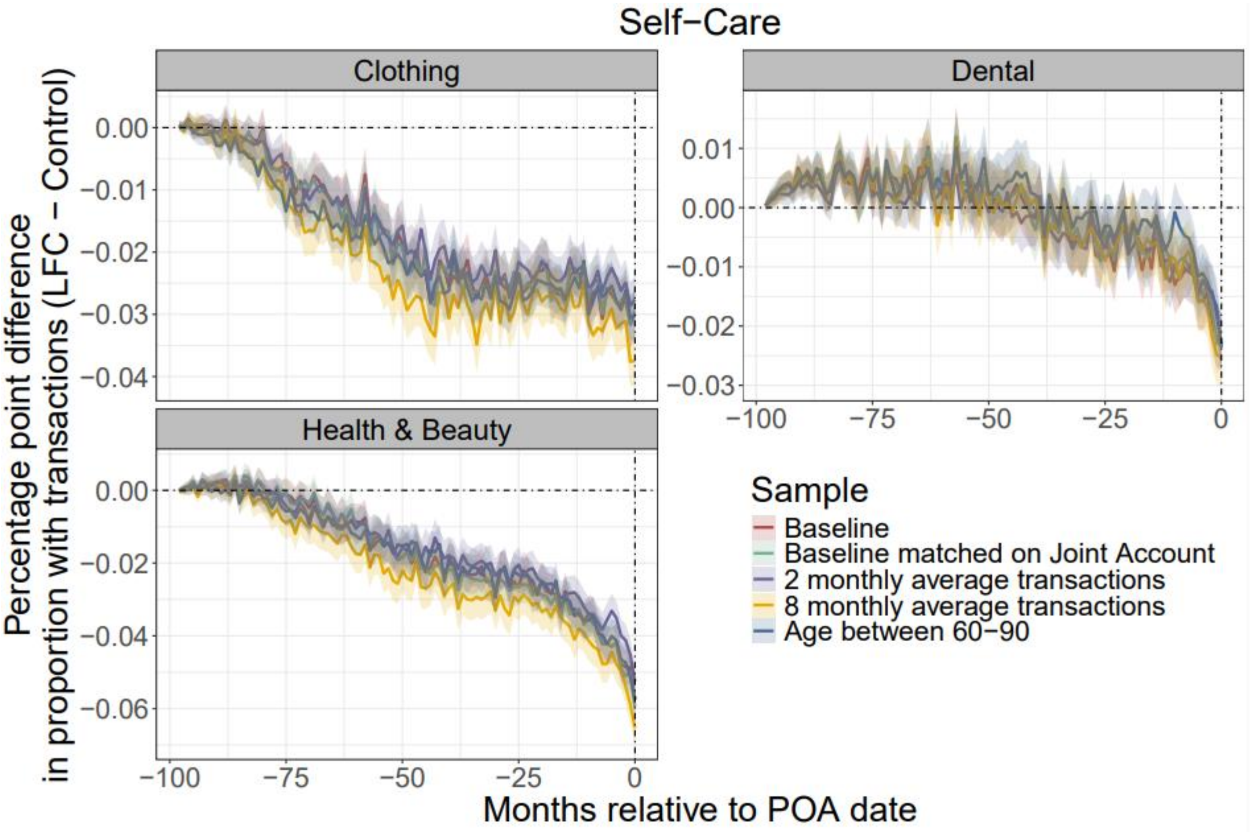

© 2025 Trendl A et al. *JAMA Network Open.*

eFigure 8. Sensitivity Results for Everyday Activities Transactional Activity

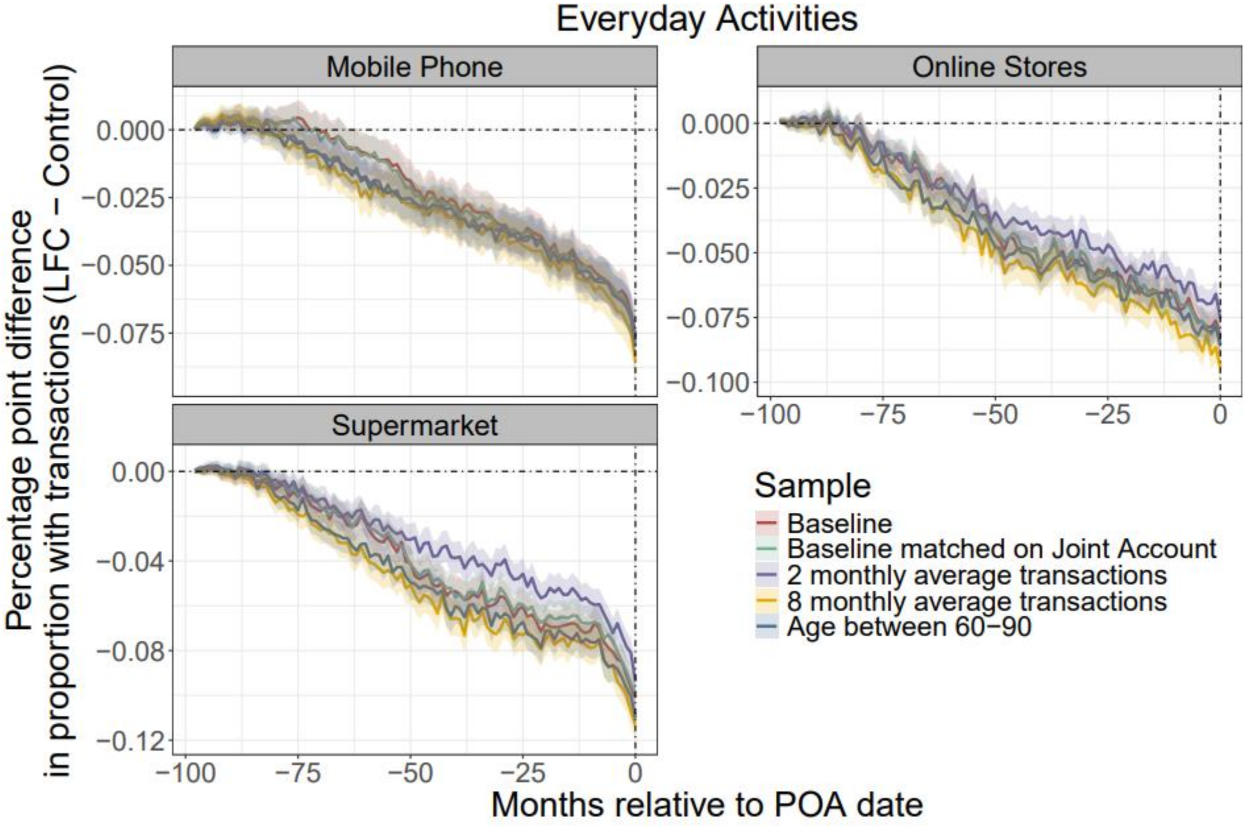

© 2025 Trendl A et al. *JAMA Network Open.*

eFigure 9. Sensitivity Results for Household Bills Transactional Activity

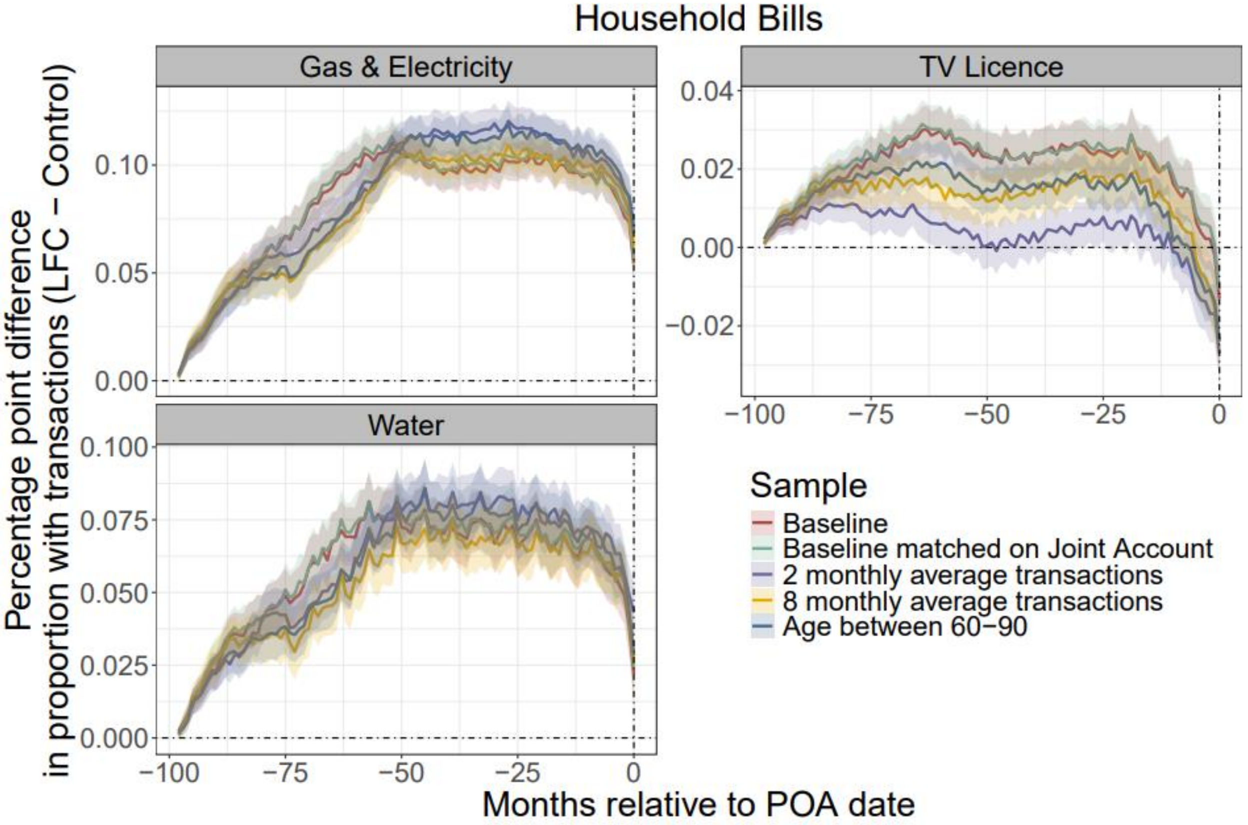

© 2025 Trendl A et al. *JAMA Network Open.*

eFigure 10. Sensitivity Results for Support Transactional Activity

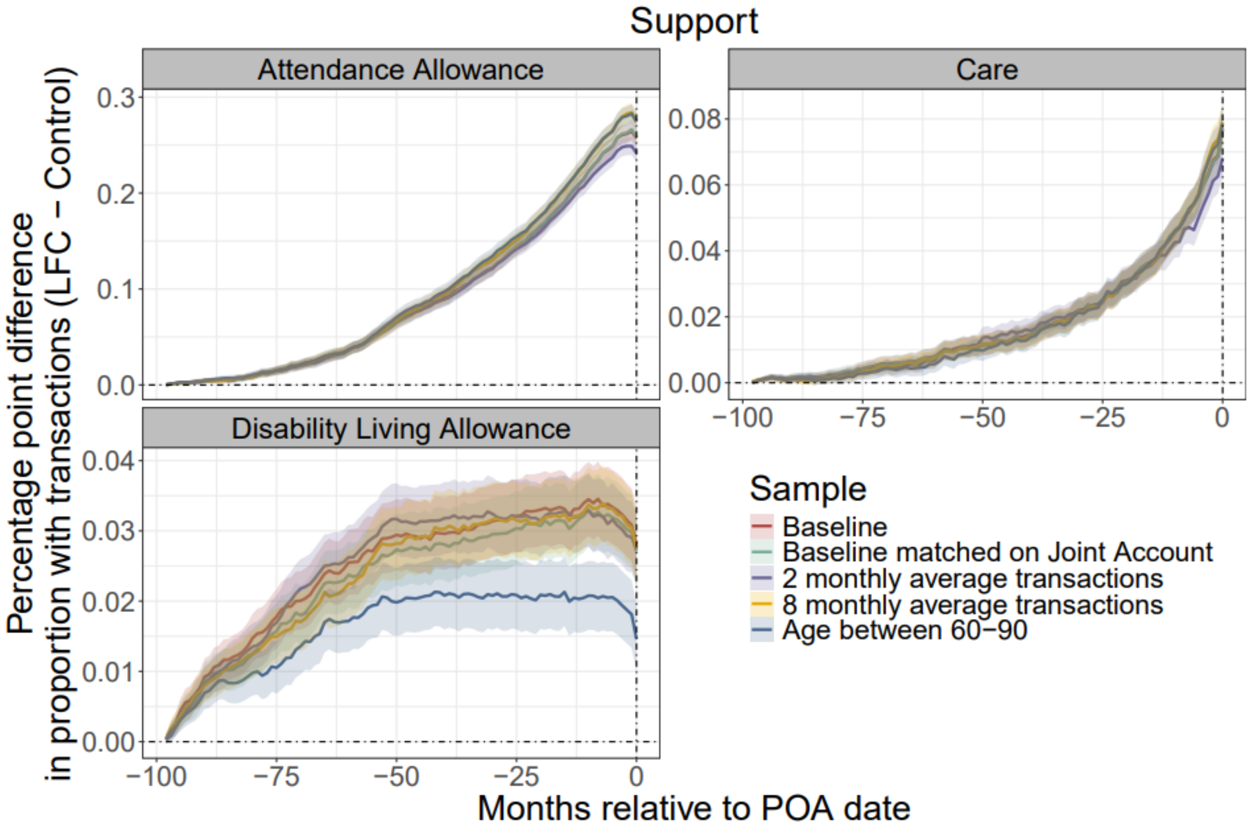

© 2025 Trendl A et al. *JAMA Network Open.*

eFigure 11. Sensitivity Results for Insurance Transactional Activity

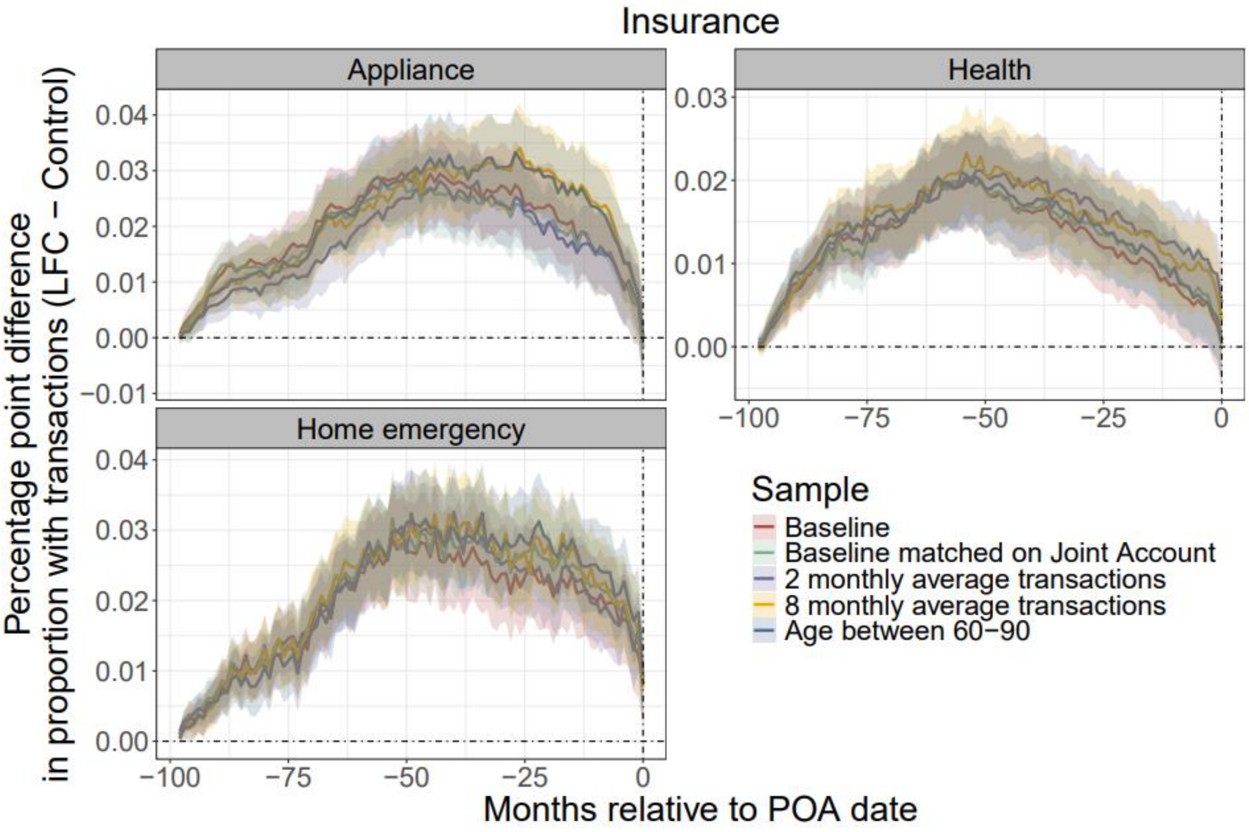

© 2025 Trendl A et al. *JAMA Network Open.*

eFigure 12. Sensitivity Results for Charity Transactional Activity

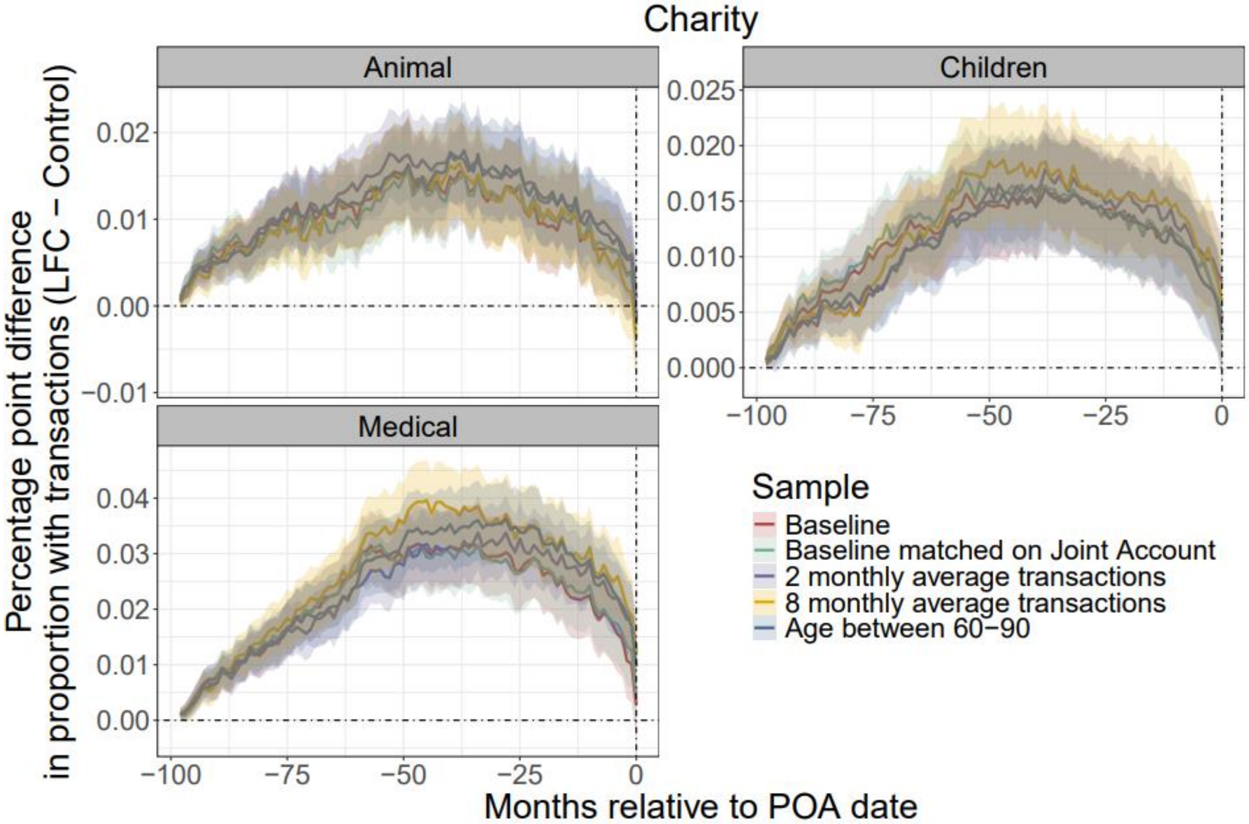

© 2025 Trendl A et al. *JAMA Network Open.*

eFigure 13. Sensitivity Results for Interactions Activity

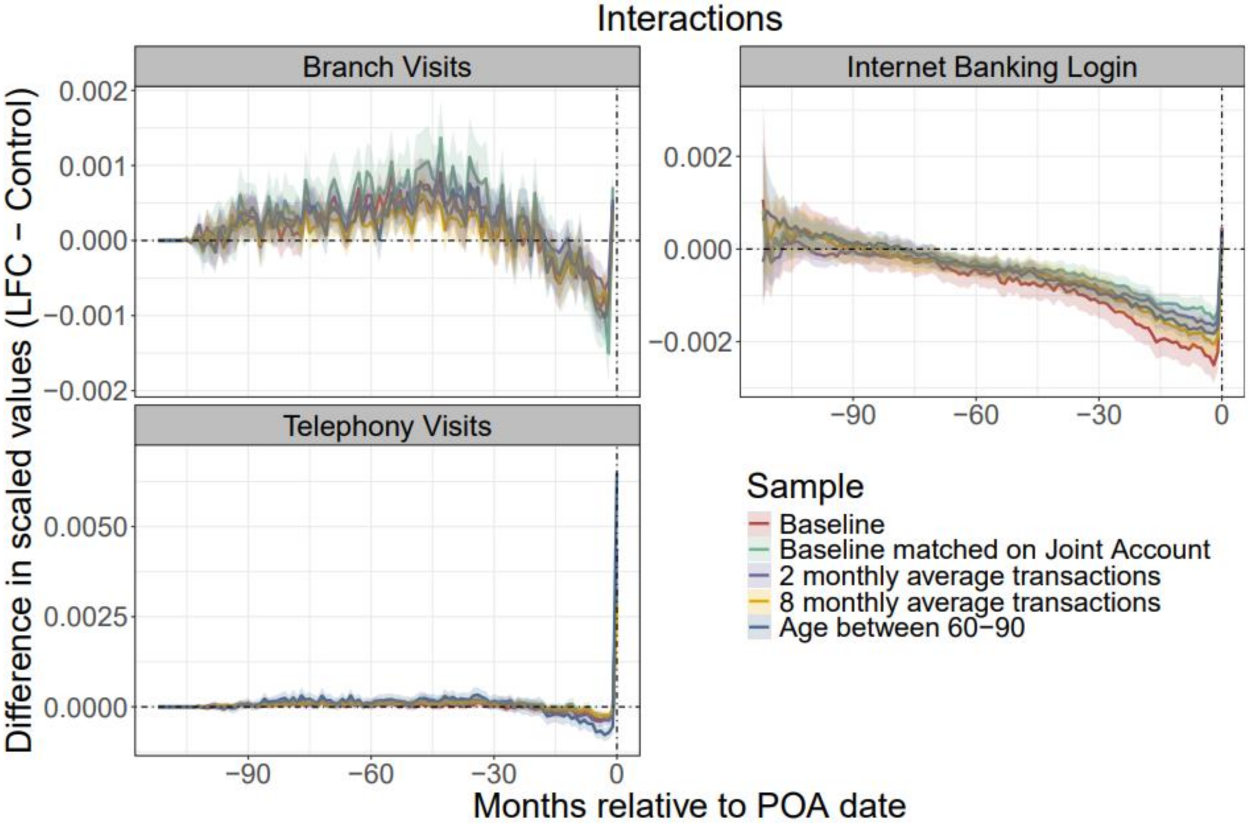

© 2025 Trendl A et al. *JAMA Network Open.*

eFigure 14. Sensitivity Results for Borrowing Activity

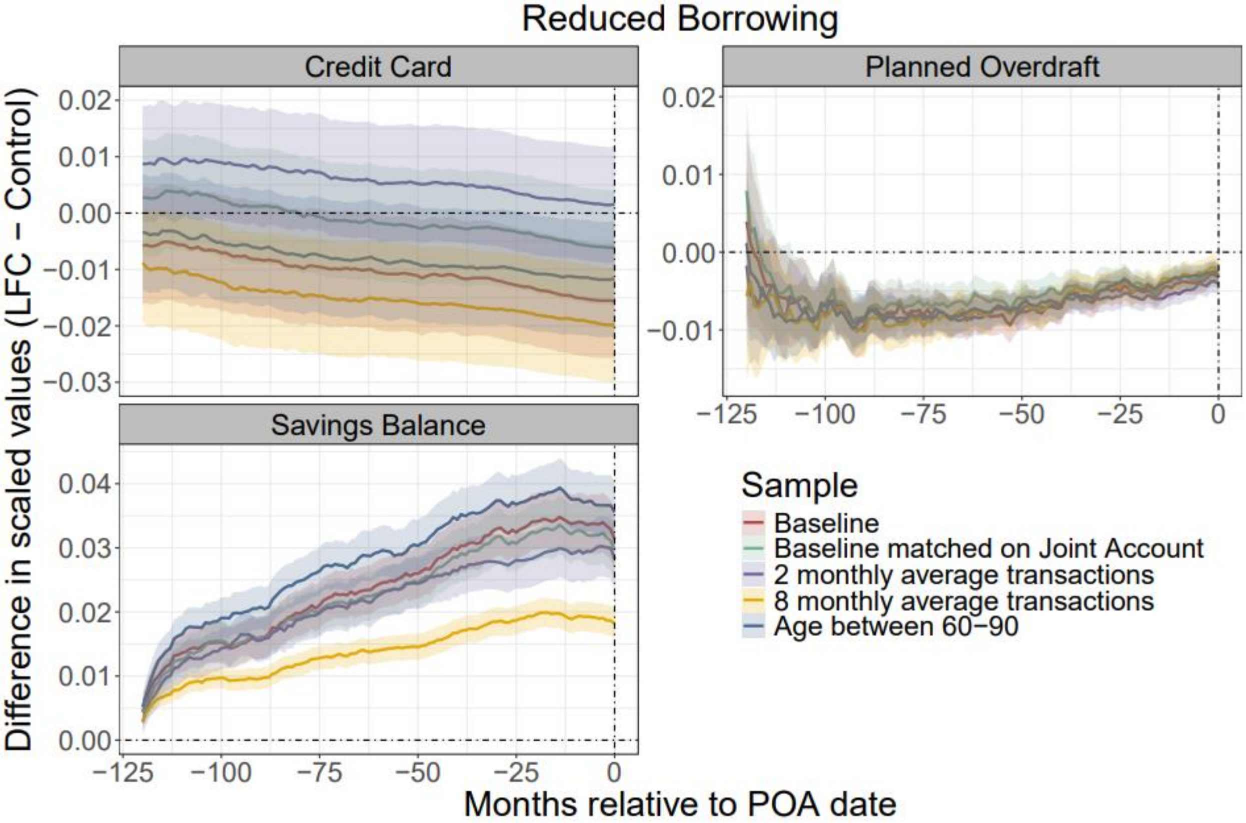

© 2025 Trendl A et al. *JAMA Network Open.*

eFigure 15. Sensitivity Results for Financial Errors

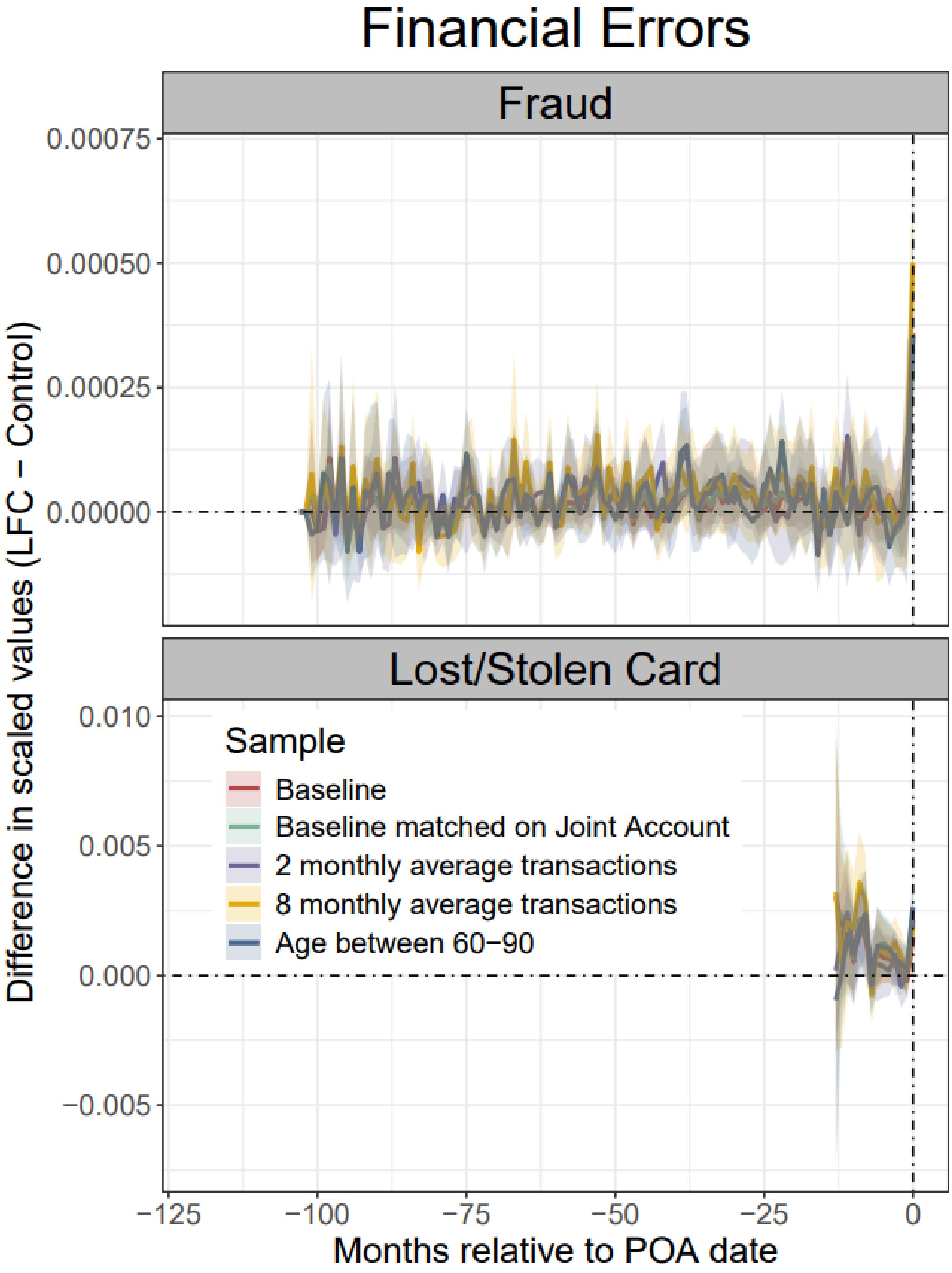

© 2025 Trendl A et al. *JAMA Network Open.*

eFigure 16. Sensitivity Results for Account Management

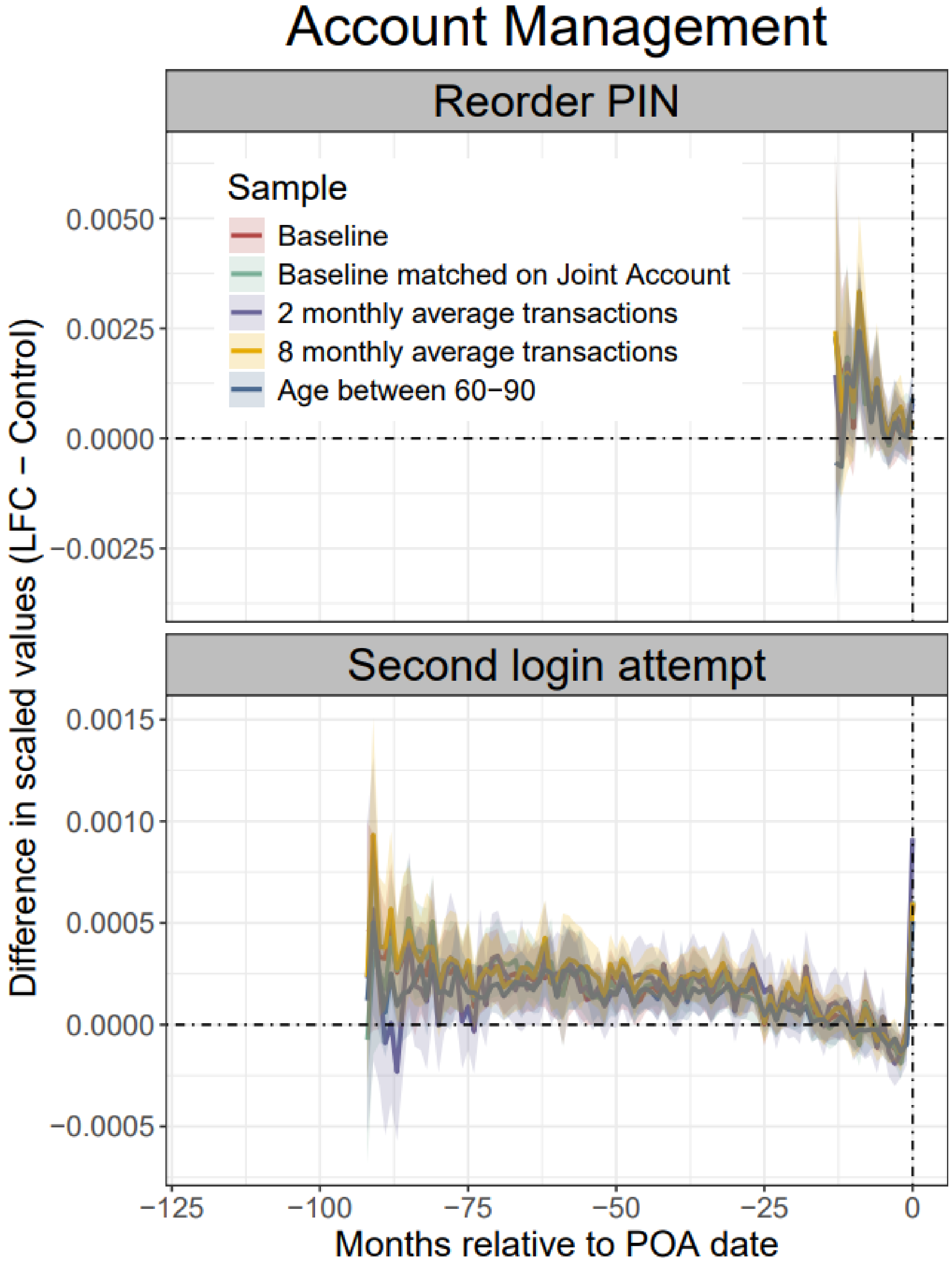

© 2025 Trendl A et al. *JAMA Network Open.*
