# Supplementary material for: Early Behavioral Markers of Loss of Financial Capacity
Source: JAMA Netw Open. 2025 Jun 13;8(6):e2515894. doi: 10.1001/jamanetworkopen.2025.15894 (PMC12166485; doi:10.1001/jamanetworkopen.2025.15894)
Supplement: Supplement 3. — Data Sharing Statement [file jamanetwopen-e2515894-s003.pdf]

Data Sharing Statement

Trendl. Early Behavioral Markers of Loss of Financial Capacity. *JAMA Netw Open*. Published June 13, 2025. doi:10.1001/jamanetworkopen.2025.15894

Data
Data available: No

Additional Information
Explanation for why data not available: The data that support the findings of this study are available from Lloyds Banking Group but restrictions apply to the availability of these data, which were used under license for the current study, and so are not publicly available. Data are available from the authors upon reasonable request and with permission of Lloyds Banking Group.
